# Retroperitoneoscopic Approach in Live Donor Left Nephrectomy: Insights From a Single-Center Case Series in the Sub-Himalayan Region

**DOI:** 10.7759/cureus.113289

**Published:** 2026-07-24

**Authors:** Karamveer Singh, Vikas Chandel, Manoj Joshua Lokavarapu, Shahbaj Ahmad, Harish Koshyari, Amit K Chauhan, Shreesh Mehrotra, Arti Rajput, Rajat Agarwal, Veena Asthana, Gurjeet Khurana

**Affiliations:** 1 General Surgery and Division of Organ Transplantation, All India Institute of Medical Sciences, Rishikesh, Rishikesh, IND; 2 General Medicine, Division of Nephrology, Himalayan Institute of Medical Sciences, Swami Rama Himalayan University, Dehradun, IND; 3 General Surgery, All India Institute of Medical Sciences, Rishikesh, Rishikesh, IND; 4 Anaesthesiology, Himalayan Institute of Medical Sciences, Swami Rama Himalayan University, Dehradun, IND; 5 Anaesthesiology, Graphic Era University, Dehradun, IND

**Keywords:** kidney transplantation, laparoscopic donor nephrectomy, living donor nephrectomy, living kidney donor, retroperitoneoscopic donor nephrectomy

## Abstract

Background and objective

Living donor nephrectomy demands a surgical approach that ensures donor safety while preserving optimal graft function. The retroperitoneoscopic approach provides direct access to the renal hilum, potentially reducing bowel manipulation and postoperative morbidity. This study aimed to evaluate the feasibility, safety, and perioperative outcomes of laparoscopic retroperitoneoscopic donor nephrectomy (LRDN) in a small case series, with a particular emphasis on donor recovery and clinical outcomes.

Methods

From November 2022 to January 2023, seven patients underwent retroperitoneoscopic donor nephrectomy (RDN) performed by a single surgeon during the surgeon’s initial independent experience, following participation in more than 100 RDN procedures at a high-volume center. All procedures were performed using a standardized retroperitoneoscopic approach. None of the donors had a history of previous abdominal surgery, and the retroperitoneoscopic approach was adopted as the standard technique. Perioperative parameters, including operative time, warm ischemia time, cold ischemia time, estimated blood loss, intraoperative complications, conversion to open surgery, length of hospital stay, and postoperative complications, were analyzed. Complications were graded according to the Clavien-Dindo classification. Donor recovery and outcomes were evaluated during the follow-up period.

Results

All seven procedures were successfully completed using the retroperitoneoscopic approach without conversion. The procedure was performed on the left side in all cases. The donor cohort comprised two males and five females, with a mean age of 46 years (range: 30-60 years). The mean operative time was 210 ± 22.9 minutes, with a mean estimated blood loss of 100.71 ± 16.94 mL. Three patients had two renal arteries. The mean warm ischemia time was 265 ± 21.41 seconds. The mean hospital stay was 3.86 ± 0.90 days. Donor recovery was uneventful in all patients, with no postoperative complications observed.

Conclusions

LRDN is a feasible and safe technique with favorable donor and graft outcomes based on the findings of this small case series. The procedure offers the advantages of minimal postoperative pain and rapid recovery, although it requires technical expertise and involves an associated learning curve.

## Introduction

Laparoscopic living donor nephrectomy is now widely regarded as the standard surgical approach for living kidney donation in most centers due to its minimally invasive nature, reduced postoperative pain, shorter hospital stays, and improved cosmetic outcomes [1-4]. This approach encompasses a spectrum of minimally invasive techniques, including pure or hand-assisted transperitoneal laparoscopy, pure or hand-assisted retroperitoneal laparoscopy, laparo-endoscopic single-site surgery (LESS), embryonic natural orifice transluminal endoscopic surgery-assisted (E-NOTES), and robotic-assisted transperitoneal or retroperitoneal approaches [5-7].

The development and refinement of minimally invasive techniques are critical to enhancing donor safety and encouraging more individuals to donate by reducing the risks and discomfort associated with the procedure. Retroperitoneoscopic donor nephrectomy (RDN) was initially described by Yang et al. in 1995. In 2000, retroperitoneoscopic live donor nephrectomy for autotransplantation and allotransplantation was described. Retroperitoneoscopic approaches have the advantage of keeping the peritoneum intact and reducing the risk of visceral organ injuries when compared with transperitoneal approaches [8-10]. Despite its relatively limited adoption compared with the transperitoneal approach, retroperitoneoscopic living donor nephrectomy is a safe, feasible, and valuable minimally invasive option. In this study, we describe our institutional experience involving seven cases of retroperitoneoscopic living donor nephrectomy, which, to the best of our knowledge, represents the first reported case series of this procedure from the sub-Himalayan region of India.

## Materials and methods

Between November 2022 and January 2023, seven patients underwent RDN performed by a single surgeon during the surgeon’s initial independent experience after having assisted in more than 100 RDN procedures at a high-volume center. All procedures were left-sided donor nephrectomies. The donor cohort comprised two males and five females. None of the donors had a history of previous abdominal surgery, and the retroperitoneoscopic approach was adopted as the standard technique.

All donors underwent a standard pretransplant evaluation, including medical, surgical, and psychological assessments. All donor evaluations were reviewed at a multidisciplinary transplant selection meeting. According to the transplant center’s policy, the left kidney was preferred for donor nephrectomy whenever anatomically and functionally suitable. Consequently, all donor nephrectomies in the present series were left-sided. Preoperative imaging was performed using CT urography with CT renal angiography. Renal function was evaluated using a technetium-99m diethylenetriaminepentaacetic acid (99mTc-DTPA) renal scan.

Donor demographic and baseline clinical characteristics, including age, sex, BMI, and nephrectomy laterality, were recorded. Additional anatomical variables, such as the number of renal arteries, renal veins, and ureters, were also documented. Perioperative parameters, including operative time, warm ischemia time, cold ischemia time, estimated blood loss, intraoperative complications, conversion to open surgery, length of hospital stay, and postoperative complications, were analyzed.

Surgical technique

**Figure 1:**
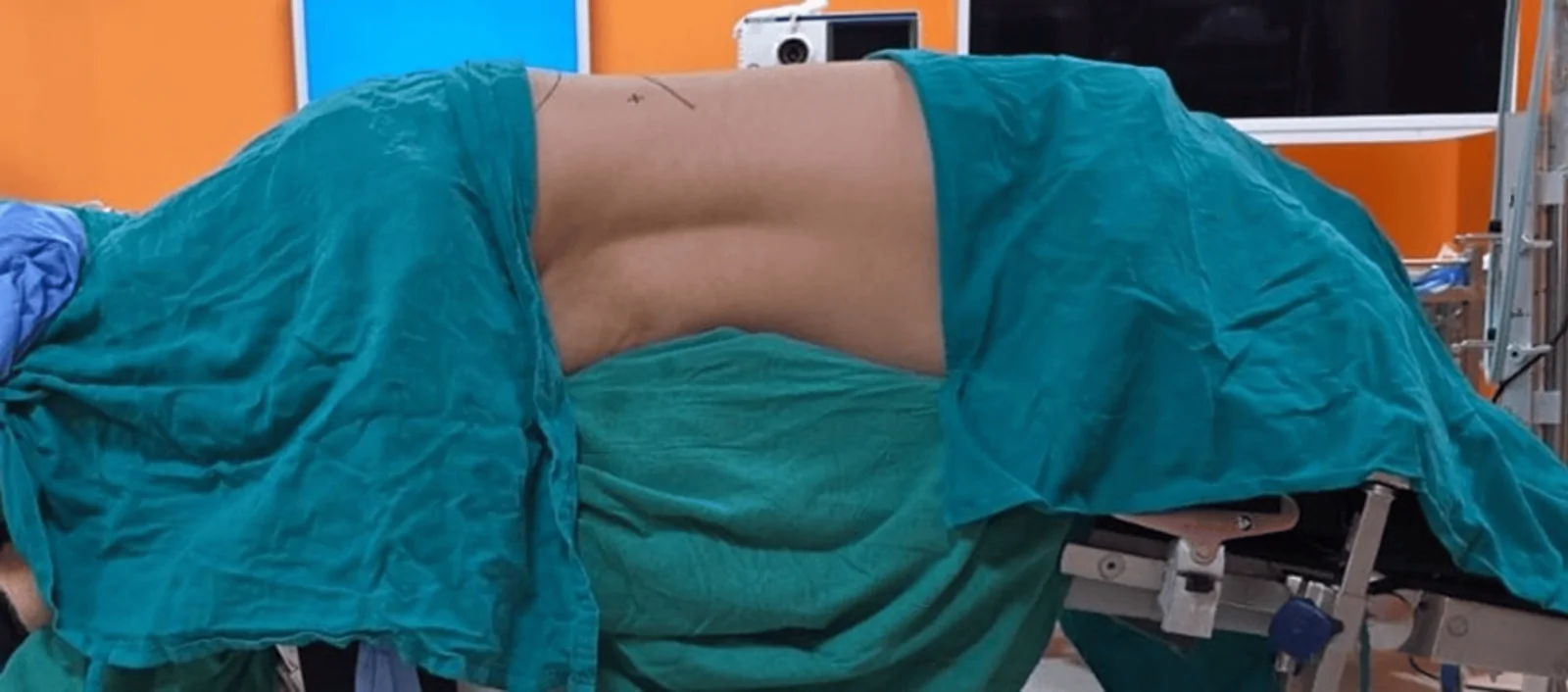
Patient positioning for left retroperitoneoscopic donor nephrectomy

The patient was catheterized and placed in a standard flank position at the edge of the operating table, with the flank muscles stretched by table flexion or through the use of a kidney bridge (Figure 1).

**Figure 2:**
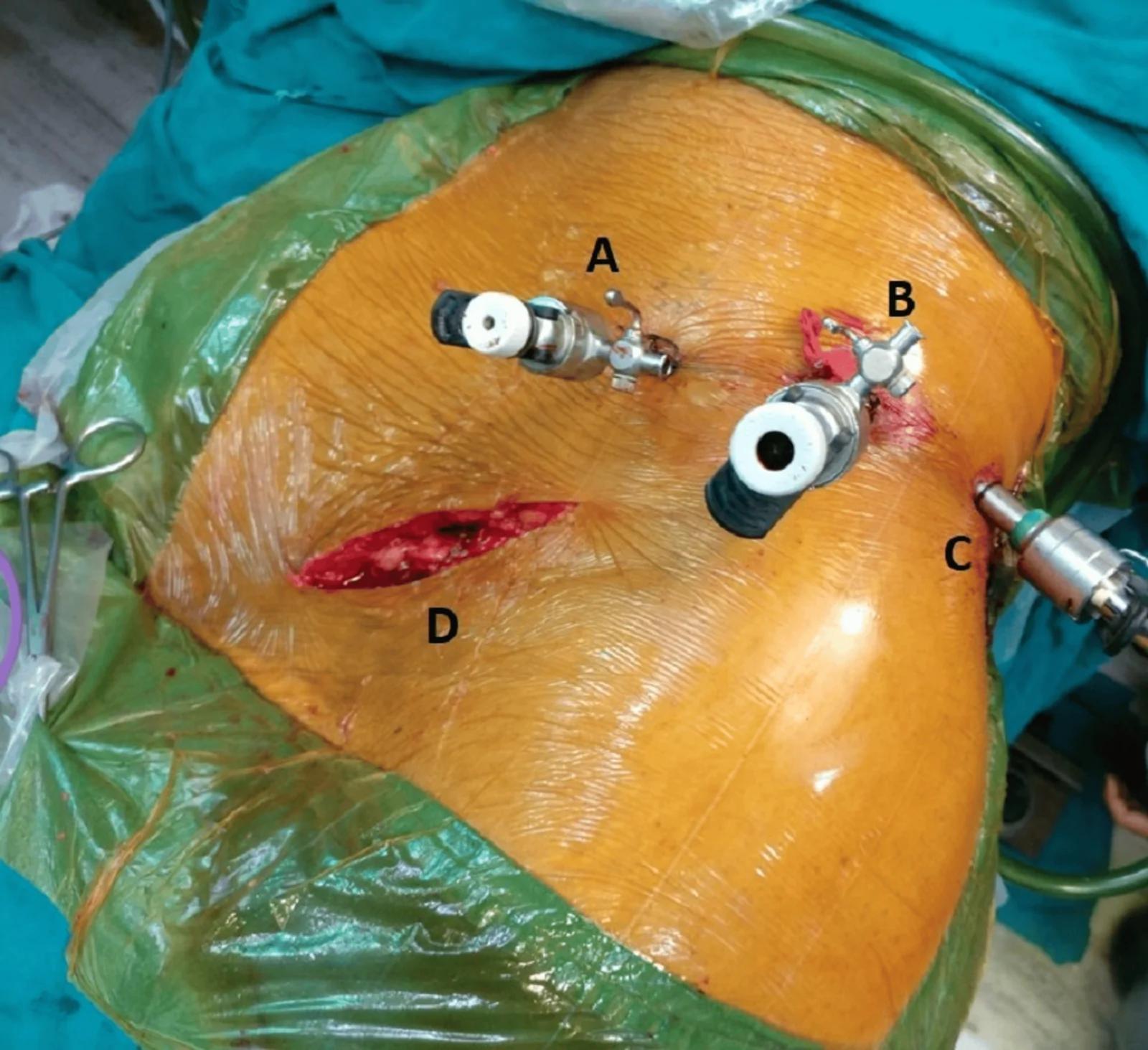
Schematic representation of trocar placement for retroperitoneoscopic donor nephrectomy A: 5 mm port; B: 10 mm port; C: 10 mm port; D: extraction incision

The patient was then securely stabilized using adhesive tape or broad straps, and the surgical field was prepared to include the tenth rib and the ipsilateral groin. Port placement was performed using the following anatomical landmarks: the paraspinous muscles, costal margin, iliac crest, and midaxillary line (Figure 2).

The first 10-mm port (B) was placed 1 cm below the intersection of the costal margin and the mid-axillary line. The second 10-mm port (C) was inserted at the renal angle, at least 3 cm away from the first port. The third 5-mm port (A) was positioned three fingerbreadths anterior to the first port, in a straight line with the other ports.

Retroperitoneal access was established through a 1.5 cm incision. The lumbodorsal fascia was incised, and perinephric fat was identified by respiratory movement and swept anteriorly off the psoas muscle using a peanut dissector. A balloon was inserted, inflated with 500 mL saline to create the working space. After confirming the extraperitoneal location by palpation, the balloon was deflated, and the space was inspected to ensure its integrity. Fixation sutures were placed to secure the access site, although these may be omitted in obese patients. A port was subsequently inserted, and carbon dioxide insufflation was initiated.

**Figure 3:**
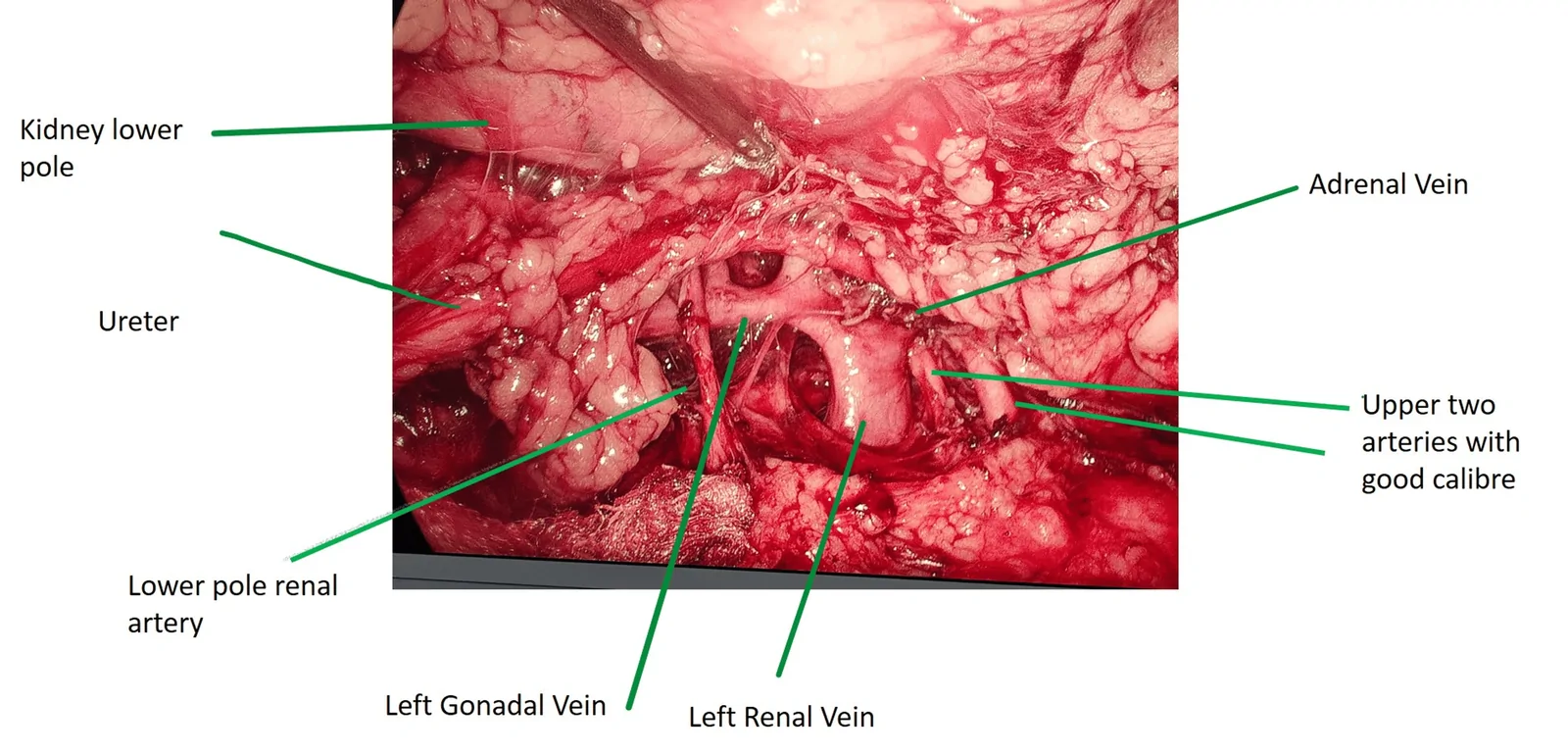
Retroperitoneoscopic view demonstrating the anatomical relationships of the kidney within the retroperitoneal space The left kidney, left renal vein, and left renal arteries are identified

The laparoscope was used to confirm peritoneal integrity and optimal port position by visualizing and then obscuring the fascial edge. The scope was rotated 30° up to identify the peritoneal reflection; digital pressure confirmed that a second 5 mm port site lay posterior to this before insertion. The laparoscope was then redirected posteriorly and inferiorly, and digital indentation was used to guide placement of the third 10-mm port; insufflation was switched here (12-15 mmHg). The psoas muscle serves as the key landmark and was kept horizontal. Gerota’s fascia was incised near the psoas muscle from the mid-ureter to the upper pole, exposing the perinephric fat, ureter, and renal hilum. The ureter was bluntly dissected while preserving the periureteric fascia, and the gonadal vein was retained or divided later. Elevation of the kidney via the 5-mm port, cushioned by posterior fat, stretches the hilum, allowing fibrolymphatic tissue to be divided and the vessels to be exposed. Variable tributaries of the left renal vein, including the adrenal, gonadal, and lumbar veins, were delineated (Figure 3).

**Figure 4:**
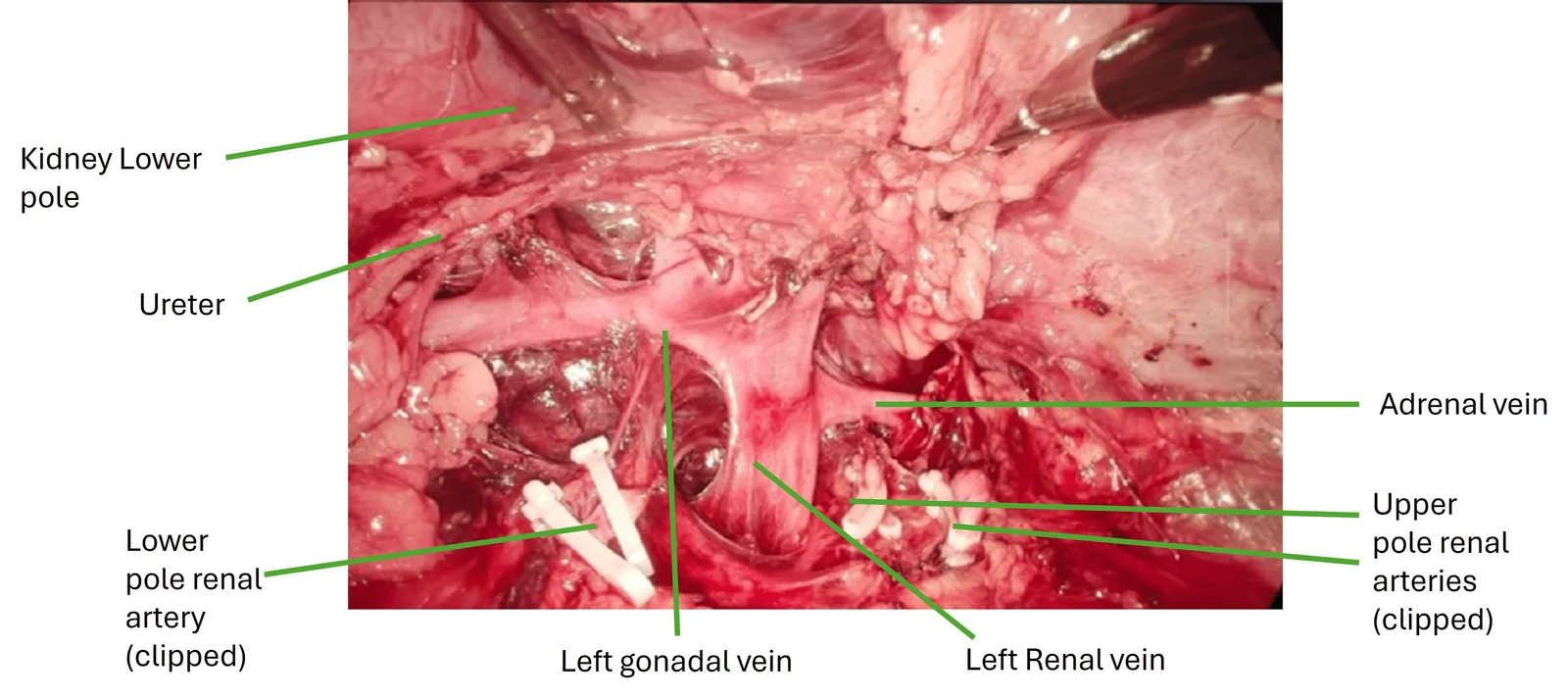
Intraoperative image demonstrating the divided renal artery with Hem-o-lok clips securely applied

The posterior lumbar vein, often adherent to the renal artery, was carefully separated and divided using clips or energy devices. The gonadal vein was managed at its confluence, either by division or preservation as a stay structure. The renal artery was exposed bluntly or sharply proximally to its aortic origin, while avoiding thermal injury. Posterior fat was excised, exposing the kidney. The kidney was mobilized anteriorly, from the upper to lower pole, and pushed posteriorly and inferiorly while avoiding arterial stretch, followed by division of the adrenal vessels. The lower pole was flipped laterally and superiorly, and dissection between the periureteric fat and peritoneum completed mobilization. The ureter was clipped distally and divided. A 6-7 cm extraction incision was made superior to the inguinal ligament; the muscles were split while preserving preperitoneal fat, and the wound was packed. The renal artery was divided and clipped using Hem-o-lok clips, ensuring a clean 2-3 mm stump. It was the operating surgeon’s standard practice to secure the left renal artery and vein with Hem-o-lok clips during left retroperitoneoscopic donor nephrectomy to maximize vascular stump length for transplantation (Figure 4).

**Figure 5:**
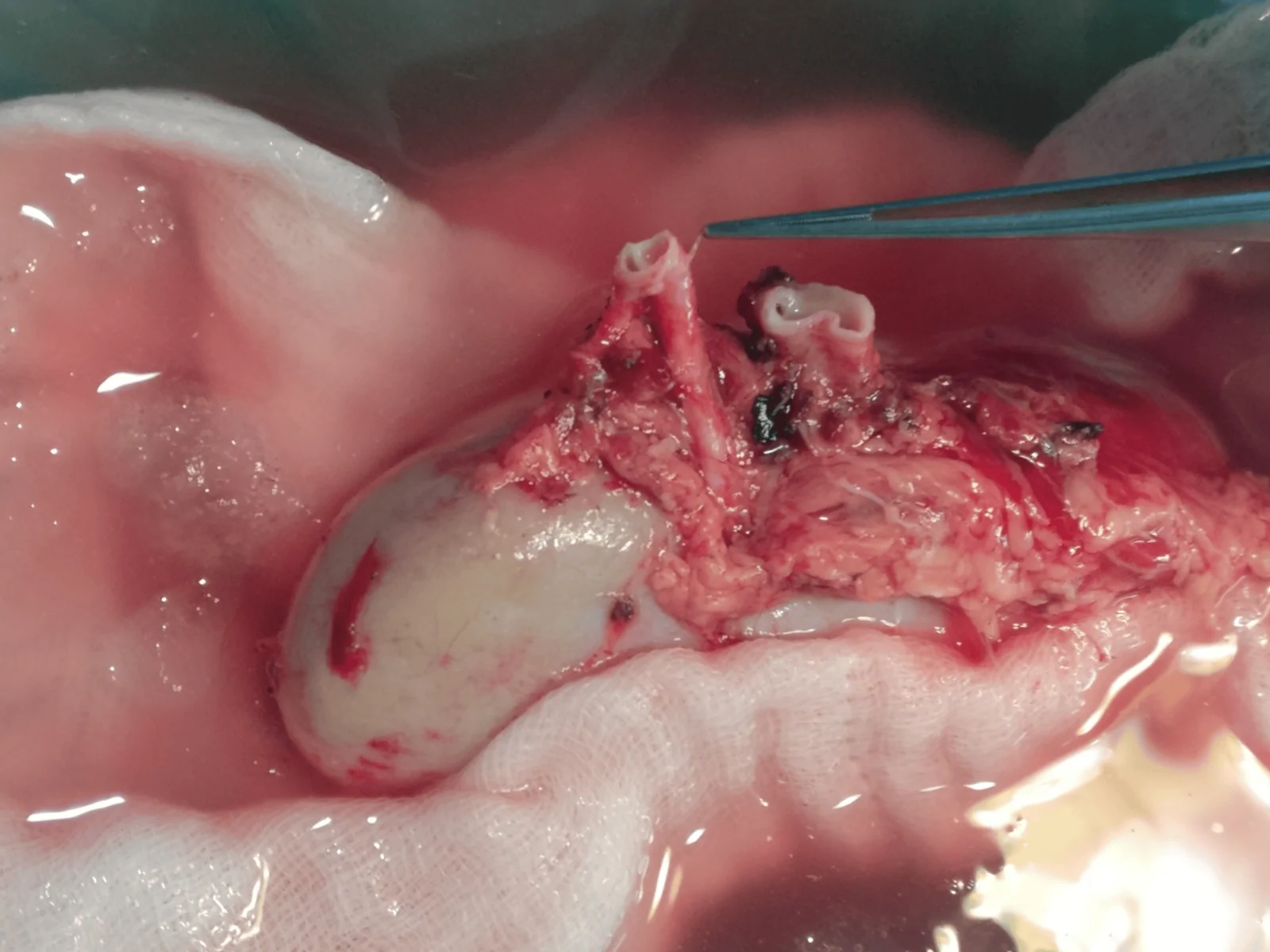
Harvested donor kidney following retroperitoneoscopic donor nephrectomy The image demonstrates intact renal parenchyma with preserved peri-hilar anatomy and adequate length of the renal artery, renal vein, and ureter before transplantation

The renal vein was stretched and divided (securing Hem-o-lok clips), and the kidney was retrieved via the incision for cold perfusion (Figure 5).

The incision was closed in layers. The pneumoperitoneum (5 mmHg) was re-established to inspect for venous bleeding; lymphatics near the artery stump were clipped. No topical hemostatic agents were used on the vascular stumps. Ports were removed under vision; the camera port sheath was closed if feasible. Skin was closed in a subcuticular fashion.

## Results

The operative procedure was completed successfully in all seven cases without conversion to open surgery. The procedure was performed on the left side in all cases. The donor cohort comprised two males and five females, with a mean age of 46 ± 12.1 years (range, 30-60 years). Preoperative donor renal function was assessed by DTPA, genitourinary imaging, and renal angiography, with a mean serum creatinine level of 0.69 ± 0.10 mg/dL and a mean glomerular filtration rate of the left kidneys of 45.87 ± 3.14 mL/min/1.73 m² (Table 1).

**Table 1:** Demographic and preoperative clinical characteristics of living kidney donors GFR: glomerular filtration rate

Patient No.	Age (years)	Gender	Pre-op serum creatinine (mg/dL)	Right kidney GFR (ml/min/1.73 m^2^)	Left kidney GFR (ml/min/1.73 m^2^)
1	48	Female	0.61	47	45
2	55	Female	0.61	39.7	41.1
3	60	Male	0.87	47.8	45.2
4	57	Female	0.6	45.5	43.7
5	34	Female	0.68	51.2	50.7
6	30	Female	0.76	45.6	47.4
7	38	Male	0.71	46	48

All donors had a single renal vein, and three donors had two renal arteries. These anatomical findings are summarized in Table 2.

**Table 2:** Operative details and perioperative outcomes of retroperitoneoscopic donor nephrectomy

Patient No.	Operative time (minutes)	Anatomy	Warm ischemia time (seconds)	Blood loss (mL)	Hospital stay (days)
1	178	Single renal artery and renal vein	240	80	3
2	190	Single renal artery and renal vein	245	80	4
3	198	Single renal artery and renal vein	250	95	4
4	222	Double renal artery and single renal vein	270	100	3
5	210	Single renal artery and renal vein	270	110	3
6	230	Double renal artery and single renal vein	280	120	5
7	242	Double renal artery and single renal vein	300	120	5

Intraoperatively, the mean operative time was 210 ± 22.9 minutes (range: 178-242 minutes), mean estimated blood loss was 100.71 ± 16.94 (range: 80-120 mL), and the mean warm ischemia time was 265 ± 21.41 seconds (range: 240-300 seconds). No major intraoperative complications were encountered. 

The mean length of hospital stay was 3.86 ± 0.90 days (range: three to five days). The urinary catheter was removed on postoperative day one. No drains were placed in any of the cases. All donors recovered satisfactorily with no postoperative complications (Table 2). All donor kidneys were successfully transplanted at our center. All recipients demonstrated immediate graft function with an uneventful postoperative recovery, and no cases of delayed graft function were observed.

## Discussion

Kidney transplantation is the definitive treatment for end-stage renal disease. Among transplant options, living donor kidney transplantation offers better recipient outcomes than deceased donor transplantation, and laparoscopic donor nephrectomy (LDN) is now considered the gold standard approach in most transplant centers. Currently, there are two main approaches for LDN: transperitoneal and retroperitoneoscopic. Retroperitoneoscopic living donor nephrectomy is a novel approach, and the majority of transplant centers prefer the transperitoneal over the retroperitoneoscopic approach [11-13].

Open donor nephrectomy has traditionally been performed through a retroperitoneal approach using a flank incision. The retroperitoneoscopic approach offers several advantages, including avoidance of intraperitoneal organ manipulation, a reduced risk of bowel-related complications such as perforation, and a lower incidence of postoperative ileus. Compared with the transperitoneal approach, it is associated with a decreased incidence of bowel adhesions and obstruction. The retroperitoneoscopic approach provides direct access to the kidney while minimizing manipulation of intraperitoneal structures and reducing the risk of injury to intra-abdominal organs. It also provides direct access to the posterior aspect of the renal hilum, allowing early identification of lumbar veins. This approach is considered safer in patients with a history of previous abdominal surgery, as it minimizes entry into the peritoneal cavity and reduces the risk of adhesiolysis-related complications. However, although the retroperitoneoscopic approach has fewer complications, it has disadvantages, including limited working space and a longer learning curve [5,12,14,15].

Across the reviewed studies, the mean operative time varied from 117 to 211 minutes for transperitoneal living donor nephrectomy and from 173 to 249 minutes for retroperitoneoscopic living donor nephrectomy. Some studies reported no significant differences in operative times between the two approaches. In our study, the mean operative time was 210 ± 22.9 minutes, which is comparable with those reported in other studies. The mean warm ischemia time reported for retroperitoneoscopic living donor nephrectomy varied between 240 and 264 seconds across the studies. The mean warm ischemia time in our study was 265 ± 21.41 seconds. The mean estimated blood loss with the retroperitoneoscopic approach ranged from 43 mL to 187 mL across several studies. The mean estimated blood loss in our study was 110.71 mL, which was comparable with previous studies [11,15-20].

The incidence of bowel injuries and splenic injuries is significantly higher with the laparoscopic transperitoneal approach than with the retroperitoneoscopic approach. The conversion rate for retroperitoneoscopic living donor nephrectomy has been reported to range from 0% to 0.7%. In our series of seven cases, there were no conversions to open procedures. There were no postoperative complications and no cases of delayed graft function among recipients [5,17,21]. The incidence of postoperative shoulder pain was significantly lower in retroperitoneoscopic living donor nephrectomy compared with transperitoneal living donor nephrectomy [15]. Patients undergoing retroperitoneoscopic living donor nephrectomy had a significantly shorter hospital stay than those undergoing the laparoscopic transperitoneal approach; however, some studies reported no difference in hospital stay between the two surgical approaches. The mean duration of hospital stay in our study was 3.86 ± 0.90 days [15,16].

Retroperitoneoscopic living donor nephrectomy is also feasible in donors with renal vein anomalies, such as circumaortic or retroaortic renal veins [13]. Several studies have shown that the laparoscopic retroperitoneal approach is associated with lower transfusion rates and lower conversion rates due to reduced vessel injury, fewer complications, and fewer cases of delayed graft function. However, patient-reported outcomes, including satisfaction with postoperative pain, scar appearance, and overall quality of life, were not significantly different between the laparoscopic retroperitoneal and transperitoneal approaches. There was also no significant difference in postoperative inflammatory marker levels between the two approaches [18,21-23].

Limitations

This study has certain limitations, including the small number of cases, its single-center design, and the absence of a control group for comparison. As these procedures represent the early experience of a single surgeon, the findings may have been affected by the initial learning curve and possible case selection bias. In addition, the limited follow-up duration did not allow for evaluation of long-term donor safety or recipient graft function.

Future directions

Advancements in laparoscopic technology, such as robotic-assisted surgery, could further enhance the safety and efficacy of laparoscopic retroperitoneoscopic donor nephrectomy (LRDN). Robotic systems offer improved dexterity and precision, potentially reducing complication rates and improving surgical outcomes. Expanding training programs for laparoscopic techniques will help disseminate best practices and improve outcomes globally. Increased access to high-quality training and mentorship can ensure that more surgeons are equipped to perform LRDN safely and effectively.

## Conclusions

This case series underscores the benefits of LRDN, demonstrating its safety, efficacy, and rapid recovery profile. The absence of complications and consistent early discharge highlight the advantages of this minimally invasive approach. Although the retroperitoneoscopic approach is a relatively novel technique and is associated with a learning curve, our experience demonstrates that it is both feasible and reproducible when performed with appropriate expertise, supporting its wider adoption and further development as an effective approach for living donor nephrectomy.

## References

[1] Greco F, Hoda MR, Alcaraz A, Bachmann A, Hakenberg OW, Fornara P (2010). Laparoscopic living-donor nephrectomy: analysis of the existing literature. Eur Urol.

[2] Wilson CH, Sanni A, Rix DA, Soomro NA (2011). Laparoscopic versus open nephrectomy for live kidney donors. Cochrane Database Syst Rev.

[3] Yuan H, Liu L, Zheng S, Yang L, Pu C, Wei Q, Han P (2013). The safety and efficacy of laparoscopic donor nephrectomy for renal transplantation: an updated meta-analysis. Transplant Proc.

[4] Serrano OK, Kirchner V, Bangdiwala A (2016). Evolution of living donor nephrectomy at a single center: long-term outcomes with 4 different techniques in greater than 4000 donors over 50 years. Transplantation.

[5] Özdemir-van Brunschot DM, Koning GG, van Laarhoven KC, Ergün M, van Horne SB, Rovers MM, Warlé MC (2015). A comparison of technique modifications in laparoscopic donor nephrectomy: a systematic review and meta-analysis. PLoS One.

[6] Breda A, Budde K, Figueiredo A (2026). EAU Guidelines on Renal Transplantation. https://d56bochluxqnz.cloudfront.net/documents/full-guideline/EAU-Guidelines-on-Renal-Transplantation-2026.pdf.

[7] Gill IS, Canes D, Aron M (2008). Single port transumbilical (E-NOTES) donor nephrectomy. J Urol.

[8] Yang SC, Park DS, Lee DH, Lee JM, Park K (1995). Retroperitoneal endoscopic live donor nephrectomy: report of 3 cases. J Urol.

[9] Gill IS, Uzzo RG, Hobart MG, Streem SB, Goldfarb DA, Noble MJ (2000). Laparoscopic retroperitoneal live donor right nephrectomy for purposes of allotransplantation and autotransplantation. J Urol.

[10] Dols LF, Kok NF, Terkivatan T (2010). Hand-assisted retroperitoneoscopic versus standard laparoscopic donor nephrectomy: HARP-trial. BMC Surg.

[11] Musquera M, Peri L, D'Anna M (2022). Outcomes after 20 years of experience in minimally invasive living-donor nephrectomy. World J Urol.

[12] Idrees M, Ng ZQ, Kuan M, Mou L (2025). Retroperitoneoscopic left live donor nephrectomy. JSLS.

[13] Tatarano S, Enokida H, Yamada Y (2019). Anatomical variations of the left renal vein during laparoscopic donor nephrectomy. Transplant Proc.

[14] S P, Krishnamoorthy V, Tyagaraj K (2025). A bidirectional cohort study to compare the outcomes of transperitoneal and retroperitoneal approaches in subjects undergoing laparoscopic live donor nephrectomy. Curr Urol.

[15] Ng ZQ, Musk G, Rea A, He B (2018). Transition from laparoscopic to retroperitoneoscopic approach for live donor nephrectomy. Surg Endosc.

[16] Hendri AZ, Soerohardjo I, Dewi KA, Danurdoro A (2023). Feasibility and safety of transvaginal specimen extraction for laparoscopic living donor nephrectomy: an Indonesian perspective compared with three different approaches. Med J Malaysia.

[17] Hirose T, Hotta K, Iwami D (2018). Safety and efficacy of retroperitoneoscopic living donor nephrectomy: comparison of early complication, donor and recipient outcome with hand-assisted laparoscopic living donor nephrectomy. J Endourol.

[18] He B, Bremner A, Han Y, Hamdorf JM (2016). Determining the superior technique for living-donor nephrectomy: the laparoscopic intraperitoneal versus the retroperitoneoscopic approach. Exp Clin Transplant Off J Middle East Soc Organ Transplant.

[19] Tay WK, Kesavan A, Goh YS, Tiong HY (2018). Right living donor nephrectomies: retroperitoneoscopic vs laparoscopic transperitoneal approach. Transplant Proc.

[20] Bachmann A, Wolff T, Ruszat R (2005). Retroperitoneoscopic donor nephrectomy: a retrospective, non-randomized comparison of early complications, donor and recipient outcome with the standard open approach. Eur Urol.

[21] Noguchi H, Shingaki K, Sato Y, Kubo S, Kaku K, Okabe Y, Nakamura M (2024). Outcomes and cost comparison of 3 different laparoscopic approach for living donor nephrectomy: a retrospective, single-center, inverse probability of treatment weighting analysis of 551 cases. Transplant Proc.

[22] Aldawsari M, Yamani A, Alqurashi NM, Althobaiti OM, Althobaiti N (2025). Comparison of transperitoneal vs. retroperitoneal laparoscopic donor nephrectomy: impact on quality of life and complications. Cureus.

[23] Gogoi D, Pal DK, Bera MK (2016). Systemic immunologic and inflammatory response after transperitoneal versus retroperitoneal laparoscopic donor nephrectomy: a prospective observational study. Saudi J Kidney Dis Transpl.

